# Global Health in the Anthropocene: Moving Beyond Resilience and Capitalism: Comment on "Health Promotion in an Age of Normative Equity and Rampant Inequality"

**DOI:** 10.15171/ijhpm.2016.151

**Published:** 2016-12-24

**Authors:** Remco van de Pas

**Affiliations:** Department of Public Health, Unit of Health Policy, Institute of Tropical Medicine, Antwerp, Belgium.

**Keywords:** Global Governance of Health, Ecology, Resilience, Sustainable Development Goals (SDGs), Capitalism

## Abstract

There has been much reflection on the need for a new understanding of global health and the urgency of a paradigm shift to address global health issues. A crucial question is whether this is still possible in current modes of global governance based on capitalist values. Four reflections are provided. (1) Ecological –centered values must become central in any future global health framework. (2) The objectives of ‘sustainability’ and ‘economic growth’ present a profound contradiction. (3) The resilience discourse maintains a gridlock in the functioning of the global health system. (4) The legitimacy of multi-stakeholder governance arrangements in global health requires urgent attention. A dual track approach is suggested. It must be aimed to transform capitalism into something better for global health while in parallel there is an urgent need to imagine a future and pathways to a different world order rooted in the principles of social justice, protecting the commons and a central role for the preservation of ecology.

Ronald Labonté’s editorial on “*Health Promotion in an Age of Normative Equity and Rampant Inequality*” is part of an important ongoing debate on the future of global governance of health.^1^ It is a debate about the fundamentals of global health challenges, more specifically on whether and why there is currently a pervasive sense of crisis in the numerous realms of the global health domain. There is much discussion about possible pathways and the governance mechanisms required to address “wicked” problems such as climate change, environmental degradation, increased income inequality, the obesity epidemic, antimicrobial resistance (AMR) and the largest human refugee streams since World War II.

There has been much reflection already in this journal, elsewhere and since over a decade on the need for a new understanding of global health, the urgency of a paradigm shift to address global health issues that display a deep connection between health (and other) sectors, and the need for thorough reform of and investment in the international organizations mandated to address global health problems, notably the World Health Organization (WHO).^2-9^ This is all punctuated with a growing understanding that global health policies are shaped by political agendas, powerful interests and inter-linked transnational networks of agencies and structures sharing like-minded norms and worldviews.^10-14^ After elaborating on why and how to arrive at a number of Sustainable Development Goal (SDG) priority goals for health, and in some cases modifying them, a crucial question is posed by Labonté in the concluding parts of the paper: “How can we tame capitalism and the predatory market logic to support human equity and (now) a livable planet? Or, if it cannot be tamed, how might capitalism be transformed into something better fit for human social and ecological survival into a 21st century?”^1^

## Different Paradigms

Two different paradigms are proposed here. The latter is inspired by the transformative thinking and holistic values behind the SDGs but remains based on the Western development model as initiated after World War II, then institutionalized via the establishment of the United Nations (UN) and the Bretton Woods institutions (International Monetary Fund and World Bank) and later the Organization for Economic Cooperation and Development (OECD). This model follows the principles of economic growth (implying an expansion of (im)material extraction, production and consumption) based on capitalism, free trade, democratization, good governance and the rule of law via cooperation between sovereign nation states.^15^ Labonté labels this approach appropriately as “a gigantic global version of Franklin Roosevelts New Deal,”^1^ basically a Neo-Keynesian investment model in green growth, decent employment, social protection and provision of public services and promoted, among others, by Nobel prize winning economists such as Paul Krugman and Joseph Stiglitz who typically advocate for fiscal expansion to foster demand in the economy. Much of the thinking behind Official Development Assistance (ODA) has been (and still is) based on providing—via bilateral and multilateral channels—some form of redistribution and leveraging investment, loans and capacity to middle- and income countries so that they can ‘grow out of poverty,’ with regulation and norms to secure public goods and advance environmental and labor rights. The hope is that eventually, a (more) democratic organization of society with respect for basic human rights follows. The SDGs follow this path, by and large: the idea is to continue, albeit in a more inclusive, deepened and universal way, the trajectory of the poverty reduction objectives of the Millennium Development Goals (MDGs), but now combined with the ecological and sustainable development consensus as outlined originally in the Rio Declaration on Environment and Development.^16^ The Ottawa Charter on Health Promotion and later also the work of the Commission on Social Determinants of Health, despite their progressive approach and demand for a reform of the dominant global economic model, both tend to advocate for change within a framework of incrementalism and inspired by a worldview that the current multilateral system of sovereign states, via balanced diplomacy, international agreements, foreign aid and policy coherence for development will eventually be able to protect the environment, secure public goods and safeguard peace and stability. As Labonté mentions, a glass half-full galvanizes more than one half-empty, and he seems more optimistic about the potential of the SDGs than about the COP21 Paris Agreement. In any case, there is an immediate requirement to analyze the political feasibility of filling the SDG glass sooner rather than later: what can really be expected from the SDG framework as a ‘politics of the improbable,’ or is instead a real paradigm shift required to tame capitalism and the predatory market logic? Observing the current political directions in major G20 economies, with global governance for public goods and international power relations being multi-polarized and gridlocked, there is even much to argue that the glass is more than half-empty. There is a sense of urgency required in imagining and constructing alternative policy pathways for just and equitable globalization for global health. I provide four reflections to complement (rather than contradict) the arguments outlined in the paper.

## Ecology Becoming a Central Value in Global Health

The former (paradigm) touches upon the centrality of ecological – centered values in any future global governance of health framework. Labonté refers to anthropogenic depravation, unequal ecological footprints, the promise of the fossil-fuel divestment movement, the 50 year-history of environmental critique as well as the importance of sustainable consumption and production patterns (while re-labelling them as the need to “consume sustainably”). Nevertheless, the focus remains in his approach, understandably, on the social and economic goals, for pragmatic reasons it appears. I would argue, instead, that the planetary ecology is so fundamentally jeopardized by the current global economic system that it must become a cross-cutting, if not central concern for all those working in global health, sooner rather than later.^17,18^

A consistent, coherent understanding of global health must be developed that integrates social and ecological objectives. Gill and Benatar note that an “alternative paradigm of ecologically health ethics is sorely needed,” one that is premised upon global solidarity and the “development of sustainability.”^19^ Global health must be properly understood as an eco-centric concept embracing the idea of a healthy people on a healthy planet, recognizing the interconnectedness of life forms and human wellbeing as well as inspired by a deep sense of responsibility and respect for our “mother earth” and future generations.^9^ Inspiration and a moral frame can be found, for example, in the ‘Laudatio Si’ encyclical letter by Pope Francis,^20^ but also in the Earth Charter.^21^ Anthony J. McMichael argued for a ‘sustainability transition’^22^ and the planetary health manifesto published two years ago stressed, rightly: “Planetary health is an attitude towards life and a philosophy for living. It emphasizes people, not diseases, and equity, not the creation of unjust societies…We need a new vision of cooperative and democratic action at all levels of society and a new principle of planetism and wellbeing for every person on this Earth.”^23^ In short, if this eco-centric approach is taken seriously we need to connect this concept with more ‘traditional’ global health objectives such as enhancing universal health coverage, reducing health inequalities, improving nutrition and access to essential medicines. If coherent, this would imply a shift from mere analysis and action on improving human and community health to a more inclusive consideration of the environmental ecosystem they are embedded in. For instance, this would shift the debate on how to deal with AMR away from the current focus on R&D of new medicines to more attention for the understanding and adaptation of the ecological context that contributes to AMR in the first place.^24^

## Fundamental Contradictions

A second reflection relates to the priority SDG goals. SDG 17, on ‘revitalizing the global partnership for sustainable development,’^25^ should be a priority to let the SDGs materialize. In essence, though, SDG 17 reveals much about the dominant political agenda lurking behind the SDGs, in spite of the transformative vision and lofty words in most of the other goals. While these are more universal, the indicators of SDG 17 still distinguish sharply between ‘developed’ and ‘developing’ countries. There is a lot of talk on ‘nudging’ countries into action and partnership with a focus on domestic resource mobilization in developing countries. In addition, “action is needed to mobilize, redirect and unlock the transformative power of trillions of dollars of private resources…long-term investments, including foreign direct investment, are needed in critical sectors, especially in developing countries.”^21^ In other words, the framing of this global partnership, combined with the ‘blended financing’ model of assistance, investment and innovation as propagated by the Addis Ababa Action Agenda on financing of development^26^ makes it evident that there is still a strong belief in the harnessing power of economic growth without really acknowledging the public ‘bads’ and the social and environmental crises it has gotten the globe into in the first place, certainly in the last decades. Although there are many believers in ‘green growth’ nowadays, the objectives of ‘sustainability’ and ‘economic growth’ present a profound contradiction.^27^ SDG 17 does not aim for sharing the responsibility between countries by mitigating the historic human rights abuses^28^ that are at the root of stark differences between high- and low-income countries, and the unforeseen, but very serious (from a health perspective) side-effects of industrialization for the integrity of the biosphere. SDG 17 does nothing fundamental to counteract the inherent and worsening instability of the current global economic system. The SDGs “offer to tinker with the global economic system in a well-meaning bid to make it all seem a bit less violent.”^27^

According to Gill ‘global governance’ is not just an analytical category but simultaneously an epistemological and strategic political project. Global health governance and the global partnership for sustainable development can be regarded as being part of a wider, though eroding, hegemonic project serving the outlook and interests of the most powerful states and affiliated actors. According to him, there is an organic crisis of global governance that raises fundamental questions about the legitimacy, ethical content and current forms of global leadership. In this sense world order and global governance can be seen as an imperial system that is predicated upon the maintenance of a fossil-fuel intensive ‘market-civilization’ and the delay of an unavoidable energy revolution as it would be accompanied with inherent power shifts.^29^ A similar reflection was made by Naomi Klein in the 2016 Edward W. Said lecture on the ongoing violence of “othering’’ in a warming world. “Climate crisis must be seen in the context of austerity and privatization, of colonialism and militarism, and of the various systems of othering needed to sustain them all. The connections and intersections between them are glaring, and yet so often resistance to them is highly compartmentalized.”^30^

Global health can be interrogated in the same way: in many instances, the ‘othering’ is perpetuated via framing it as ‘just’ a problem of developing countries with scarce resources and poor governance in a context of fragility combined with a limited awareness by communities of their health situation and lack of access to the innovative wonders of modern medicine. Global power structures that maintain inhumane health situations, such as those that became evident during the 2014-2015 Ebola outbreak in West-Africa, remain neglected.^31-33^ In our times of ‘deep’ economic globalization based on deregulated finance and free trade (plus the inherent democratic deficits), there is only a marginal policy space for nation states who find themselves in a fiscal race to the bottom to develop progressive social and ecological policies. Admittedly, there is ‘a rise of the rest’ and nation states and emerging economies, like the BRICS, have chosen alternative development modalities.^3^ However, in our (now) multipolar world, these powers favor new financial institutions, known as the “new non-Western financial model,” over investing in the leadership, finance, and strategic directions of existing global health governance institutions such as the WHO, UNAIDS or the Global Fund.^4^ Political scholars have come to the conclusion that there is a gridlock in global governance domains,^34^ although there is debate whether this also applies to global health.^35^ Studies from the ecological field clearly indicate the instability and limits of the current carbon-constrained capitalist growth model and the planetary boundaries.^12,13^ In short, the analysis above provides a sobering view on (the expectations for) the financing to be generated via the global partnership on development. The glass will probably only remain half-full or, in these times of increasing nationalism and a backlash against globalization, might even be emptied further. Consequently, if the world remains within this capitalist model, even of a more “sustainable” and “inclusive” kind, grand global health claims such as “a world converging within a generation”^36^ or the prediction that the current trend of economic growth will continue and provide the fiscal space in LMICs to employ 18 million extra health workers required to attain the health objectives of the SDGs by 2030^37^ remain a gamble.

## The Status Quo of Resilience and Multi-stakeholder Governance

The third and fourth reflection follow the thinking above that the SDGs might (only) be locked in the status quo of global governance rather than display the transformative shift they are usually associated with. Reflections on resilience and multi-stakeholder governance are dealt with on their own merits in longer debates and papers^38,39^ so they are only touched upon briefly here. In his article, Labonté alludes to the distraction of the resilience discourse and I can’t agree more with him. Although resilience capabilities might be one of more desirable outcomes of health systems strengthening^40^ the normative thinking behind much of the current resilience discourse is that crises are permanent and that individuals, thus, have to be permanently prepared for the worst. Evans and Reid note: “The real tragedy for us is the way the (resilience) doctrine forces us to become active participants in our own de-politicisation… it even demands a certain exposure to the threat before its occurrence so that we can be better prepared. Resilience as such appears to be a form of immunization.”^41^ By internalizing resilience as the main principle of dealing with insecurity, it becomes part of self-policing. Neocleous concludes “In so doing resilience shapes our political imagination and thereby cuts off alternate political possibilities.”^42^ This includes cutting of the moral imagination (the ability to imagine oneself in the shoes of others) that can enable to alter one’s outlook and actions significantly.^43^

The Ebola outbreak in Western Africa catalyzed further development of the Global Health Security Agenda, a partnership representing governments, academia and the private sector, built on the value of resilience and the notion that “Our connectedness…poses serious challenges with implications for our health security and for the stability and security of our populations.”^44^ The very valid question remains whose interests and whose security are predominantly being served by this agenda, and whether alternative models of overcoming infectious diseases epidemics can be developed.^45^

Multi-stakeholder governance is mentioned by Labonté as one of the core contradictions of the SDGs. Indeed, without enforced regulation for the public good, this sort of governance might continue to be a smokescreen for legitimizing the powerful actors and interests that contributed most to the current economic and ecological crises. While in general there is a need to strengthen forms of deliberative democracy beyond the nation state,^46^ including in institutions dealing with global health, a lot needs to improve on the output legitimacy (accountability, transparency effectiveness) and certainly input legitimacy (deliberation and representation) of global health and sustainable development regimes.^47^ Research on the development of the health SDG goal has indicated limited participation of local communities in setting priorities for this goal and the governance gap between the global policy-makers and the ‘target groups.’^48^ Proper regulation, the management of conflicts of interest and a strong democratic framework to govern global health programs all require close attention in the implementation of the SDGs. This should not merely be about the international organizations itself and their engagement with corporate actors, but also touch upon the politics and agency of philanthropic organizations, academia and civil society.^49-52^

## Moving Forward

In conclusion I would argue, somewhat similarly, for a ‘dual track’ approach as formulated by Labonté. We have to continue with our aim to gradually transform capitalism into something better *while in parallel* we should be well aware that we need to move beyond resilience and capitalism fast, and thus, imagine a future and pathways to a different world order rooted in the principles of social justice and protecting the commons with a central role for the preservation of ecology. Despite my reservations described above, multiple health crises and the cosmopolitan window of opportunity they create^53^ could perhaps trigger a momentum within the SDG framework to have more global public goods for health universally (co)-financed, such as basic public health functions and universal

health coverage. It must, however, be noted that current representations of the right to health in the SDGs are insufficient and superficial, because they do not explicitly link commitments or right to health discourse to binding treaty obligations for duty-bearing nation states or entitlements by people.^54^

If the crises become deep enough there will be a stronger push for global redistribution mechanisms like an international tax regime or the need to regulate the harms of our overheated consumerist societies.^55^ More importantly we should allow for moral and political imagination and conceptualize alternative views of organizing societies. Recent publications on the ‘Politics for the Anthropocene’^56^ and ‘Realistic Cosmopolitanism’^57^ hint towards the inherent shared responsibility required to govern civilization, the environment and global risks. Thinking along the lines of the End of capitalism^58^ the Basic Income Earth Network^59^ as well as the Degrowth and Divest movement,^60^ Indigenous principles of ‘Buen vivir’^61^ and citizens reclaiming the common goods^62^ all provide elements of hope. The global health community should hence not only pursue this important debate in academic journals or at global policy meetings but participate actively in societal movements and debates to help drive a real (and clearly much needed) paradigm shift. This trajectory is deeply political and risky. In the words of Eduardo Galeano: *“I advance two steps, it goes two steps backward. I take ten steps and the horizon moves ten steps forward. No matter how far I walk, I will never reach it. What is the use of utopia? That’s its use: to help us walk.”*^63^ Yet, we owe it to the next generations.

## Ethical issues

Not applicable.

## Competing interests

Author declare​s​ that he has no competing interests.

## Author’s contribution

RvdP is the single author of the paper.
